# Olfactory Receptor Neurons for Plant Volatiles and Pheromone Compounds in the Lucerne Weevil, *Sitona discoideus*

**DOI:** 10.1007/s10886-020-01160-y

**Published:** 2020-02-12

**Authors:** Kye Chung Park, Mark R. McNeill, David M. Suckling, C. Rikard Unelius

**Affiliations:** 1 The New Zealand Institute for Plant and Food Research Limited, Christchurch, 8140 New Zealand; 2 AgResearch Limited, Private Bag 4749, Christchurch, 8140 New Zealand; 3 School of Biological Sciences, University of Auckland, Auckland, 1142 New Zealand; 4 Faculty of Health and Life Sciences, Linnaeus University, 391 82 Kalmar, Sweden

**Keywords:** Host-plant volatiles, Olfactory sensilla, Pheromone, Single-sensillum recording, 4-methyl-3,5- heptanedione, 5-hydroxy-4-methyl-3-heptanone

## Abstract

Antennal olfactory receptor neurons (ORNs) for pheromone-related and plant volatile compounds were identified and characterized in the lucerne weevil, *Sitona discoideus* (Gyllenhal), using the single sensillum recording technique. Our study using five pheromone-related compounds and 42 plant volatile compounds indicates that *S. discoideus* have highly specialized ORNs for pheromone and plant volatile compounds. Different groups of ORNs present in both males and females of *S. discoideus* were highly sensitive to 4-methylheptane-3,5-dione (diketone) and four isomers (*RR*, *RS*, *SR* and *SS*) of 5-hydroxy-4-methylheptan-3-one, respectively. Our results also indicate that male *S. discoideus*, using the sensory input from antennal ORNs, can distinguish both diketone and the *RR*-isomer from others, and *RS*- and *SS*-isomers from others, although it was unclear if they can distinguish between *RS*-isomer and *SS*-isomer, or between diketone and the *SR*-isomer. It also appeared that female *S. discoideus* could distinguish between *RS*-isomer and *SS*-isomers. The antennae of *S. discoideus* thus contain sex-specific sets of ORNs for host- and non-host plant volatile compounds. Both sexes of *S. discoideus* have highly sensitive and selective ORNs for some green-leaf volatiles, such as (*Z*)-3-hexenol and (*E*)-2-hexenal. In contrast, male antennae of *S. discoideus* house three distinct groups of ORNs specialized for myrcene and (*E*)-β-ocimene, 2-phenylethanol, and phenylacetaldehyde, respectively, whereas female antennae contain three groups of ORNs specialized for (±)-linalool and (±)-α-terpineol, myrcene and (*E*)-β-ocimene, (±)-1-octen-3-ol, and 3-octanone. Our results suggest that *S. discoideus* use a multi-component pheromone communication system, and a sex-specific set of ORNs with a narrow range of response spectra for host-plant location.

## Introduction

*Sitona discoideus* (Gyllenhål) (Coleoptera: Curculionidae), commonly called the lucerne weevil, was first reported in New Zealand in 1974 when larvae were found feeding on burr medic (*Medicago polymorpha* L.) near Napier in the North Island (Esson 1975). Originally from Morocco, it was believed that the weevil had been accidentally introduced from Australia via lucerne (*M. sativa* L.) hay used for horse fodder (Esson 1975). In January 1976, *S. discoideus* was first recorded in Canterbury and Otago in New Zealand (Somerfield and Burnett 1976). However, the weevil had probably established in the South Island much earlier than indicated by Somerfield and Burnett’s 1976 paper (Wood 1980). In 1982, *S. discoideus* was reported from most parts of New Zealand where lucerne and annual medicago species were grown (Kain and Trought 1982).

*Sitona discoideus* has a univoltine life cycle in New Zealand (Goldson et al. 1984; Sue et al. 1980), with adult emergence from the soil in December–January (summer), followed by a period of vigorous feeding by the adults, during which time adults reach flight competency and then fly to aestivation sites located along hedge rows and fence posts (Frampton 1987; Goldson et al. 1984). Aestivation lasts for 7–8 weeks during which time the adults remain reproductively immature and do not feed. Post-aestivatory return flights usually commence in the latter part of March and peak in mid-April (autumn) (Goldson et al. 1984). The return to the lucerne is marked by reproductive maturation between the months of March and May (Goldson et al. 1984). The biology in New Zealand is similar to that observed in the Mediterranean habitat which is considered to be the species center of origin (Aeschlimann 1979).

Another *Sitona* species, *S. obsoletus* (Gmelin) considered to be synonymous with *S. lepidus* and *S. flavescens* (Coleoptera: Curculionidae) (Löbl and Smetana 2013), was first discovered in the North Island of New Zealand in March 1996 (Barratt et al. 1996). An initial survey indicated that the weevil may have been present much earlier, as it was widely established and therefore eradication was impracticable. Of Palearctic origin, *S. obsoletus* is usually univoltine in northern hemisphere countries (Levesque and Levesque 1994; Markkula 1959), but because of New Zealand’s temperate maritime climate it has two generations a year (Gerard et al. 2010). It has a strong preference for white clover (*Trifolium repens* L.) (Crush et al. 2010; Murray and Clements 1994), but elsewhere has also been reported as a pest of red clover (*T. pratense* L*.*) (Murray et al. 2007). The weevil is now widely established across New Zealand (Hardwick et al. 2016). Both *S. discoideus* and *S. obsoletus* commence egg laying in autumn (March) in New Zealand which continues through winter into early spring (November). Adults of both species feed on the foliage and the larvae feed on the root nodules and roots. Feeding by larvae of both *S. discoideus* and *S. obsoletus* can cause significant yield losses in lucerne (Goldson et al. 1988) and white clover (Care et al. 2000; Gerard et al. 2007), respectively.

Studies have shown that 4-methyl-3,5-heptanedione seemingly acts as a general aggregation pheromone for *S. lineatus* L. (Blight et al. 1984) and that it is also attractive to several other *Sitona* species (Toshova et al. 2009; Toth et al., 1998). We have reported earlier that male *S. discoideus* emit both 4-methyl-3,5-heptanedione (major) and (4*S*,5*S*)-5-hydroxy-4-methyl-3-heptanone (minor), during the autumnal post-aestivatory flight period (Unelius et al. 2013). Isomers of the latter compound are called sitophilure, as its (4*S*,5*R*)-isomer is the major aggregation pheromone component for two beetles of the *Sitophilus* genus, the rice weevil and the maize weevil (Schmuff et al. 1984; Walgenbach et al. 1987).

The studies on the olfactory receptor neurons (ORNs) provide clues to potential species-specific sets of ORNs and their potential use for host and mate location in insects. In a recent study, we have identified ORNs for pheromone-related compounds and plant volatiles for *S. obsoletus* (Park et al. 2013). In the present study, we identified ORNs in *S. discoideus*, and compared the ORN profiles between these two closely related weevil species. These two species appeared to have different needs for their host-plant location during their adult life span, since the lucerne weevil is in need of long-range detection of lucerne fields, whereas the *S. obsoletus* always stays in the clover field. Our hypothesis was that we would find some differences in the ORN setup between the two species as a reflection of their different behavioral ecology. To examine this, we carried out single-sensillum recordings on the antennae of male and female *S. discoideus*, using a range of pheromonal compounds and host- and non-host plant volatiles.

## Methods and Materials

### Insects

Adults of *S. discoideus* used in the experiments were collected in the Canterbury region of New Zealand. Males and females were distinguished based on the shape of the ventrite (Blight et al. 1984) and kept in separate containers with fresh-cut lucerne.

### Test Compounds and Odor Presentation

**Fig. 1:**
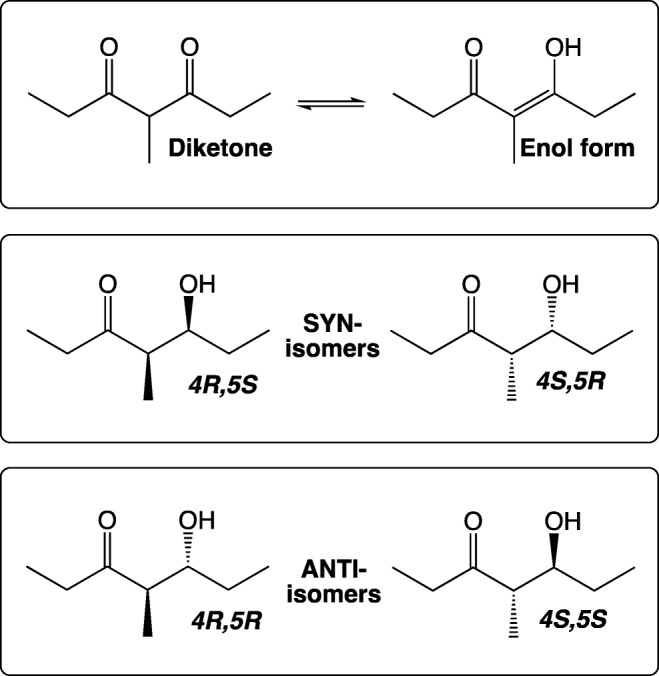
4-Methyl-3,5-heptanedione (‘diketone’) and its enol form, and the four stereoisomers of sitophilure, 5-hydroxy-4-methylheptan-3-one, pheromone components in the *Sithophilus* and *Sitona* genera

Five pheromone-related compounds, 4-methyl-3,5-heptanedione (‘diketone’) and the four stereoisomers (RR, RS, SR and SS) of 5-hydroxy-4-methyl-3-heptanone (Fig. 1), and 42 host or non-host plant volatile compounds were used as stimuli in the single-sensillum recording (SSR) (Table 1). At least 17 of the plant volatile compounds investigated for SSR activities are present in lucerne, red clover and white clover (Buttery et al. 1984; Core et al. 1994; Figueiredo et al. 2007; Kicel et al. 2010; Kigathi et al. 2009; Landon et al. 1997; Tava and Pecetti 1997). The source, purity and presence in lucerne and clovers of the test compounds are shown in Table 1. The plant volatile compounds were purchased from commercial sources, and the pheromone-related compounds were synthesized as described in Bohman and Unelius (2009). Each compound was dissolved in hexane as a 50 ng/μL solution, except the green-leaf volatile (GLV) compounds (Table 1), which were prepared in mineral oil at the same concentration. The test compounds were divided into nine groups (Table 1), and the mixture solution of each group was also prepared in hexane (or in mineral oil for the group Mix-GLV) at a concentration of 50 ng/μL for each compound in the group. Hexane or mineral oil was used as the solvent control stimulus. Serial dilutions of some compounds were also prepared in hexane or mineral oil for measuring dose responses of ORNs. One μg dose of each compound was chosen as a test dose based on a previous study on *S. obsoletus* (Park et al. 2013). Further dilutions were prepared with two compounds, 1-octen-3-ol and 3-octanone, to measure dose-responses to these compounds as they displayed very strong responses at 1 μg dose.

**Table 1:** Test compounds for the single-sensillum recording study of *Sitona discoideus* and their source, purity, and presence in lucerne and clovers

Mixture group	Compound	Plant^a^	Purity^b^	Source
Mix-P	4-Methylheptane-3,5-dione (diketone)		90%^c^	synthesized
	(4*S*,5*S*)-5-Hydroxy-4-methylheptan-3-one (SS)		>98% (97% ep)	synthesized
	(4*S*,5*R*)-5-Hydroxy-4-methylheptan-3-one (SR)		>98% (95% ep)	synthesized
	(4*R*,5*S*)-5-Hydroxy-4-methylheptan-3-one (RS)		>98% (96% ep)	synthesized
	(4*R*,5*R*)-5-Hydroxy-4-methylheptan-3-one (RR)		>98% (99% ep)	synthesized
Mix-GLV	1-Hexanol	L^1^, R^3^	99%	Aldrich
	(*E*)-2-Hexen-1-ol	L^1^,	96%	Aldrich
	(*Z*)-2-Hexen-1-ol	R^4^	95%	Aldrich
	(*Z*)-3-Hexen-1-ol	L^1,2,^ R^4^, W^5^	98%	Aldrich
	Hexanal	L^1^,	98%	Aldrich
	(*E*)-2-Hexenal	L^1,2^, R^4^, W^5^	98%	Aldrich
	Hexyl acetate		99%	Aldrich
	(*Z*)-3-Hexenyl acetate	L^1,2,6^, R^4,7^	98%	Aldrich
	2-Heptanone		99%	Aldrich
Mix-A	1-Nonanol		98%	Fluka
	Ethyl (2*E*,4*Z*)-2,4-decadienoate		98%	Bedoukian
	(*E*)-β-Farnesene	L^6^, R^3,4,7^	98%	Bedoukian
	(−)-Caryophyllene	R^3,4^	98.5%	Sigma
	(−)-Germacrene-D		40%	Treat & Co
Mix-B	(±)-Limonene	L^1^, R^7^, W^5^	97%	Merck
	Myrcene		95%	Aldrich
	(*E*)-β-Ocimene	L^1^, R^4,7^	70%	Fluka
	(±)-α-Pinene		99%	Aldrich
Mix-F	(±)-β-Pinene		99%	Aldrich
	α-Phellandrene		95%	Aldrich
	γ-Terpinene		97%	Aldrich
	1,8-Cineole (eucalyptol)		98%	Aldrich
	(±)-Citronellal		95%	Aldrich
	(±)-α-Terpinyl acetate		90%	Aldrich
Mix-C	(±)-α-Terpineol		90%	Aldrich
	Nerol	L^6^	96%	Aldrich
	Geraniol		98%	Aldrich
	(±)-Linalool	L^1^, R^7^, W^5^	97%	Aldrich
	2-Phenylethanol	L^1^, R^3^, W^5^	99%	Fluka
Mix-D	Benzaldehyde	L^1^, R^3^, W^5^	99.5%	Aldrich
	Citral (geranial + neral)		96%	Aldrich
	Phenylacetaldehyde		90%	Aldrich
Mix-E	Benzyl acetate		99%	Aldrich
	Diethyl malonate		99%	Aldrich
	Geranyl acetate		98%	Aldrich
	Isobutyl phenylacetate		98%	Aldrich
	Methyl benzoate		99%	Aldrich
	Methyl phenylacetate		99%	Aldrich
	Neryl acetate		96%	Aldrich
Mix-G	(±)-1-Octen-3-ol	W^5^, R^7^	98%	Aldrich
	3-Octanone	L^1,2^, W^5^	98%	Aldrich

^a^Plants emitting the compounds: L (lucerne, *Medicago sativa*); R (red clover, *Trifolium pratense*); W (white clover, *T. repens*)

^b^Chemical purity (ep: enantiomeric purity)

^c^The diketone is in equilibrium with its enol tautomer (c. 10% according to GC)

^1^Tava and Pecetti. (1997), ^2^ Landon et al. (1997), ^3^ Figueiredo et al. (2007); ^4^ Buttery et al. (1984); ^5^ Kicel et al. (2010); ^6^ Core et al. (1994) ^7^ Kigathi et al. (2009)

Presentation of test chemicals to the insect antennae was similar to previous studies (Park and Baker 2002, Park and Hardie 2004, Park et al. 2013). A 20-μL aliquot of each test solution was applied onto a piece (5 × 30 mm) of filter paper (Whatman No 1, USA), and the filter paper strip was inserted into a glass Pasteur pipette (146 mm, Fisher Scientific, USA) after being evaporated for 10 s in air. The tip of the pipette was inserted into a small hole (2 mm diameter, 10 cm from the outlet to the antennae) in a glass main airflow tube with a continuous, charcoal-filtered and humidified airflow (600 mL/min) over the antennal preparation. A 0.1-s pulse of charcoal-filtered airflow (10 mL/s) was injected through the wide end of the Pasteur pipette odor cartridge for stimulation, using an electronic airflow controller (CS-55, Ockenfels Syntech GmbH, Kirchzarten, Germany). The wide end of the Pasteur pipette was covered with a piece of aluminum foil when not in use, to reduce evaporation. Each odor stimulus cartridge was used fewer than 10 times.

### Single-Sensillum Recording

A weevil was mounted on a Plasticine® block with U-shaped thin copper wire restraints, and each antenna was further fixed using fine copper wires. Then the preparation was positioned in the middle of the charcoal-filtered and humidified main airstream. A fine tip (tip diameter < 10 μm) glass electrode (0.86 mm ID, A-M Systems Inc., USA) filled with 0.1 M KCl was inserted into a membranous part of the abdomen to serve as the reference electrode. An electrochemically sharpened tungsten electrode (tip diameter < 0.1 μm) was used as a recording electrode and the position of the electrodes was controlled with micromanipulators (Leitz, Germany; Sutter Instruments, USA). An Ag-AgCl junction was used to maintain electrical continuity between the reference electrode and the ground input of a high-input impedance headstage preamplifier (Ockenfels Syntech GmbH, Kirchzarten, Germany). The AC signals through the preamplifier were further amplified, digitized at 12,000/s sampling rate, and processed with a PC-based signal processing system (IDAC-4, Syntech, Buchenbach, Germany) and software (Autospike 32, Ockenfels Syntech GmbH, Kirchzarten, Germany).

**Fig. 2:**
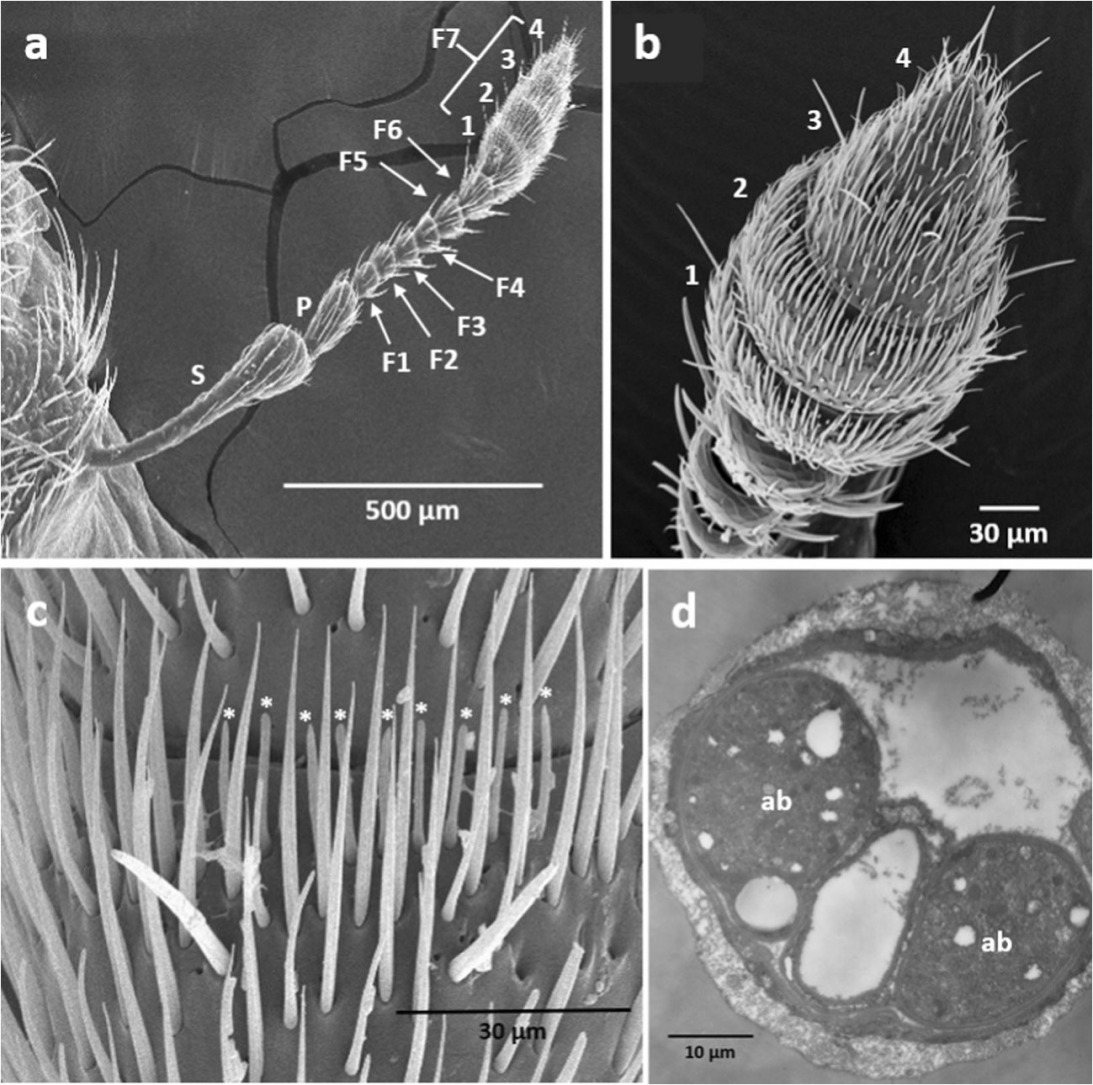
Antennal morphology of *Sitona discoideus*. **a**: The seven-segment antennal flagella (F1 - F7) bear sensilla, with the majority of the sensilla being located at the terminal club (**b**). **c**: A number of basiconic sensilla (asterisks) are located at the circumferential regions around the distal area of the first three (**b**: 1 ~ 3), forming a so-called ‘sensory band’, in four subsections of the terminal club (F7). **d**: The cross-section of a flagellum observed by transmission electron microscopy shows that two axon bundles (*ab*) run inside the length of the flagella segments. S: scape; P: pedicel

Once a stable contact was made between the electrodes and a sensillum, showing spontaneous firing of action potentials, the antenna was stimulated with a series of nine mixtures of test compounds (Table 1). If any electrophysiological response was observed after the stimulation with mixtures, the antenna was further stimulated with the individual compounds contained in the mixture eliciting responses. The order of testing chemicals was made at random. The time interval between successive stimulation was approximately 30 s. When a response lasted for a long time (e.g. >30 s), sufficient time was allowed until spontaneous activity returned to background levels before re-stimulation. Sensilla on the three circumferential sensory band regions on the club-shaped terminal flagella segment (Fig. 2 a, b, c) were mainly investigated from 15 females and 17 males in this study. The responsiveness of ORNs was analyzed by comparing the number of action potentials before and after odor stimulation. In each recording, the number of action potentials for 1 s after odor stimulation was subtracted by the average number of action potentials for 1 s before the stimulation. Then, the averaged numbers (*n* = 2~27 depending on sensillum types) of the subtracted values were classified into seven different categories of ORN response strength. The ORNs were then classified into different types using an algorithm with step-by-step analysis according to their response profiles across the test compounds (Table 2).

**Table 2:** An algorithm for classifying the types of sensilla and olfactory receptor neurons (ORNs) (PM: pheromone; PL: plant volatile) in *Sitona discoideus*. The types of sensilla and ORNs were systematically classified according to their electrophysiological responsiveness to different groups of test compounds

Step	Grouping principle	Resulting groups
1	Show spontaneous activity	Go to 2
	No spontaneous activity	Discard (no ORNs)
2	Respond to pheromone mixture	Go to 3 (**Type PM**)
	Respond to plant volatile	Go to 4 (**Type PL**)
	No response to pheromone mixture or plant volatile mixtures	Non-responsive ORNs
3	Strong response to 4-methylheptane-3,5-dione: > 40 Hz	Go to 5
	Weak or fair response to 4-methylheptane-3,5-dione: < 40 Hz	Go to 6
4	Respond to a specific group of compounds (< 10 compounds)	Go to 7
	Respond to a broad range of compounds (≥ 10 compounds)	This type not found
5	No or weak (<20 Hz) response to RR-isomer^a^	**Go to 8**
	Fair (> 30 Hz) response to RR-isomer^a^	**Type M-PM-B**
6	Fair (> 30 Hz) response to RR, RS, SR and SS-isomers^a^	**Type F-PM-C**
	No (<10 Hz) response to RS, SR and SS-isomers^a^	**Type M-PM-C**
7	Fair or strong response to green leaf volatiles only	Go to 9
	No or weak response to green leaf volatiles	Go to 10
8	Strong responses to RS, SR and SS-isomers in males	**Type M-PM-A**
	Strong responses to RS, SR and SS-isomers in females	**Type F-PM-A**
	No or weak responses to SS-isomer	**Type F-PM-B**
9	Strong response to *Z*3–6:OH and fair response to *E*2–6:Ald in males	**Type M-PL-A**
	Strong response to *Z*3–6:OH and *E*2–6:Ald in females	**Type F-PL-A**
	Strong response to *Z*3–6:OH; No response to *E*2–6:Ald in males	**Type M-PL-B**
	Strong response to *Z*3–6:OH; No response to *E*2–6:Ald in females	**Type F-PL-B**
10	Strong response to phenylacetaldehyde	**Type M-PL-C**
	Strong response to myrcene and (*E*)-β-ocimene in males	**Type M-PL-D**
	Strong response to 2-phenylethanol	**Type M-PL-E**
	Strong response to (±)-linalool	**Type F-PL-C**
	Strong response to (±)-citronellal	**Type F-PL-D**
	Strong response to myrcene and (*E*)-β-ocimene in females	**Type F-PL-E**
	Strong response to 1-octen-3-ol and 3-octanone	**Type F-PL-F**

^†^Increase of the number of spikes by >10 spikes/s after the stimulation was regarded as a ‘response’

^‡^Weak response (<20 spikes/s); Fair response (≥ 20 and < 50 spikes/s); Strong response (≥ 50 spikes/s)

^a^RR: (4*R*,5*R*)-5-Hydroxy-4-methylheptan-3-one; RS: (4*R*,5*S*)-5-Hydroxy-4-methylheptan-3-one;

SR (4*S*,5*R*)-5-Hydroxy-4-methylheptan-3-one; SS (4*S*,5*S*)-5-Hydroxy-4-methylheptan-3-one

## Results

### Location of Olfactory Sensilla on the Antennae

The club-shaped terminal flagella segment of the antennae of male and female *S. discoideus* (Fig. 2a) consisted of four sub-segments (Fig. 2a and b). The circumferential region of the first and second sub-segments contained a number of short basiconic-type olfactory sensilla (Fig. 2c asterisk), forming a so-called “sensory rim” (Fig. 2b and c). Two main axon bundles run inside the length of the antenna (Fig. 2d, ab). Our SSR studies measured the responsiveness of ORNs present in these sensilla.

### Overall Response Profile of Antennal Olfactory Sensilla in *S. discoideus*

The ORNs observed in *S. discoideus* showed spontaneous firing of action potentials at 15.62 ± 0.83 spikes/s (mean ± SE, *n* = 29, minimum 7, maximum 25) and 13.24 ± 1.59 spikes/s (mean ± SE, *n* = 41, minimum 1, maximum 45) in pheromone-related ORNs of males and females, respectively, and at 3.44 ± 1.21 spikes/s (mean ± SE, *n* = 16, minimum 0, maximum 15) and 2.95 ± 1.12 spikes/s (mean ± SE, *n* = 22, minimum 0, maximum 23) in plant volatile-related ORNs of males and females, respectively. Mineral oil used as a solvent for green leaf volatiles elicited no activity in any of the ORNs tested. In contrast, hexane used as a solvent for all other test compounds elicited significant responses from some ORNs. In the *S. discoideus* antennae examined, 58 sensilla (37 in males and 21 in females) were found to contain ORNs responsive to pheromone-related compounds (Table 3), and 143 sensilla (72 in males and 71 in females) contained ORNs for plant volatile compounds (Table 4). The sensilla containing ORNs responsive to pheromone-related compounds were classified into six types (Table 3), and the sensilla containing ORNs responsive to plant volatile compounds into eleven types (Table 4), based on the response spectra to the test compounds of the ORNs housed in these sensilla. Co-compartmentalized ORNs responsive to both pheromone-related compounds and plant volatile compounds were not found in *S. discoideus* in our study (Table 5). Six types of sensilla appeared to contain ORNs, indicated as ‘Unknown’ in Table 5, showing no responses to any of the compounds tested, but showing spontaneous firing of action potentials (Table 5).

**Table 3:** Types of sensilla containing olfactory receptor neurons (ORNs) responsive to pheromone-related compounds, identified in males and females of *Sitona discoideus*, and their responsiveness to five pheromone-related compounds. Different colors indicate different ORNs co-compartmentalized in the same sensillum

Sex	Male			Female		
Type	M-PM-A	M-PM-B	M-PM-C	F-PM-A	F-PM-B	F-PM-C
N	21	8	8	6	8	7
Diketone	**+++++++**a	**++++**a	**++**a	**+++++++**a	**+++++++**a	**+++**a
RR	**+**c	**+++**ab	**++**a	**++**bc	**+**c	**++++**a
RS	**+++++**b	**++**d	**+**b	**++++**bc	**++**c	**++++a**
SR	**+++++**b	**+++**bc	**+**b	**+++++**b	**++++**b	**++++**a
SS	**++++++**b	**++**cd	**+**b	**+++++**ab	**+**c	**++++**a
Hexane^2^	**+**c	**+**e	**+**b	**+**c	**+**c	**+**b

^†^The number of ‘+’ indicates the responsiveness of each type of ORNs for the corresponding pheromone-related compounds. The average number of spikes: + (< 10 spikes/s); ++ (< 20 spikes/s); +++ (< 30 spikes/s); ++++ (< 40 spikes/s); +++++ (< 50 spikes/s); ++++++ (< 60 spikes/s); +++++++ (≥ 60 spikes/s)

^‡^Diketone: 4-Methylheptane-3,5-dione; RR: (4*R*,5*R*)-5-Hydroxy-4-methylheptan-3-one; RS: (4*R*,5*S*)-5-Hydroxy-4-methylheptan-3-one; SR: (4*S*,5*R*)-5-Hydroxy-4-methylheptan-3-one; SS: (4*S*,5*S*)-5-Hydroxy-4-methylheptan-3-one

^2^Hexane: solvent blank control

Different letters indicate that means (mean number of spikes/s after stimulation) are significantly different within a column (Tukey HSD, *p* = 0.05)

**Table 4:** Types of sensilla containing olfactory receptor neurons (ORNs) responsive to plant volatile compounds identified in males and females of *Sitona discoideus* and their responsiveness to 42 plant volatile compounds. Different colors in M-PL-A, F-PL-A and F-PL-F indicate different ORNs co-compartmentalized in the same sensillum

Sex	Male					Female					
Type	M-PL-A	M-PL-B	M-PL-C	M-PL-D	M-PL-E	F-PL-A	F-PL-B	F-PL-C	F-PL-D	F-PL-E	F-PL-F
N	15	27	12	15	3	14	18	5	2	14	18
Hexane^1^					**+**	**+**		**+**	**+**		
Mineral oil^1^											
Hexane^2^											
1-Hexanol	**++++**	**++++**				**++++**	**+++**				
(*E*)-2-Hexen-1-ol	**++++++**	**+++**				**++++**	**++++**				
(*Z*)-2-Hexen-1-ol	**+++++++**	**+++++**				**++++**	**++++**				
(*Z*)-3-Hexen-1-ol	**+++++++**	**+++++++**				**++++**	**++++**				
Hexanal	**++**					**++**					
(*E*)-2-Hexenal	**+++**					**++++**					
Hexyl acetate	**+**					**+**	**+**				
(*Z*)-3-Hexenyl acetate	**+**						**+**				
2-Heptanone	**+**					**+**					
1-Nonanol											
Ethyl decadienoate^3^											
(*E*)-β-Farnesene											
(−)-Caryophyllene											
(−)-Germacrene-D											
(±)-Limonene				**++**						**+**	
Myrcene				**+++++++**						**+++**	
(*E*)-β-Ocimene				**+++++++**						**++++**	
(±)-α-Pinene				**++**						**+**	
(±)-β-Pinene									**+**		
α-Phellandrene											
γ-Terpinene									**+**		
1,8-Cineole (eucalyptol)											
(±)-Citronellal									**+++++++**		
(±)-α-Terpinyl acetate									**+**		
(±)-α-Terpineol					**+**			**++++**			
Nerol					**+**			**+++**			
Geraniol					**+**			**+**			
(±)-Linalool								**+++++++**			
2-Phenylethanol					**+++++++**			**++**			
Benzaldehyde			**+**								
Citral (geranial + neral)			**+**								
Phenylacetaldehyde			**++++**								
Benzyl acetate											
Diethyl malonate											
Geranyl acetate											
Isobutyl phenylacetate											
Methyl benzoate											
Methyl phenylacetate											
Neryl acetate											
(±)-1-Octen-3-ol											**+++++**
3-Octanone											**+++++**

^1^Hexane and mineral oil were solvent blanks

^2^Hexane was dissolved in mineral oil and used as a stimulus

^3^Ethyl decadienoate: Ethyl (2*E*,4*Z*)-2,4-decadienoate

^†^The number of ‘+’ indicates the responsiveness of each type of ORNs for the corresponding pheromone-related compounds. The average number of spikes: + (< 10 spikes/s); ++ (< 20 spikes/s); +++ (< 30 spikes/s); ++++ (< 40 spikes/s); +++++ (< 50 spikes/s); ++++++ (< 60 spikes/s); +++++++ (≥ 60 spikes/s)

**Table 5:** Co-compartmentalized olfactory receptor neurons (ORNs) identified in *Sitona discoideus* antennae. The ORNs were distinguished by comparing the amplitude of action potentials generated by the corresponding stimulus volatile compounds. ‘Unknown’ indicates that a separate ORN was co-compartmentalized in the same sensillum, but the ORN exhibited no responses to any compounds tested in this study

Sensillum type	ORN	Active compound^a^
M-PM-A	I	RS, SS
	II	Diketone, SR
M-PM-B^b^	I	Diketone, RR, RS, SR, SS
M-PM-C	I	RR
	II	Diketone
M-PL-A	I	*Z*2–6:OH, *E*2–6:OH, *Z*3–6:OH
	II	*E*2–6:Ald
M-PL-B	I	*Z*3–6:OH
	II	Unknown^c^
M-PL-C	I	Phenylacetaldehyde
	II	Unknown^c^
M-PL-D	I	Myrcene, (*E*)-β-ocimene
	II	Unknown^c^
M-PL-E	I	2-Phenylethanol
	II	Unknown
F-PM-A	I	RS, SS
	II	Diketone, SR
F-PM-B	I	RS
	II	Diketone, SR
F-PM-C^b^	I	Diketone, RR, RS, SR, SS
F-PL-A	I	*Z*2–6:OH, *Z*3–6:OH
	II	*E*2–6:Ald
F-PL-B^b^	I	*E*-6:OH, *Z*2–6:OH, *Z*3–6:OH
F-PL-C	I	(±)-Linalool, (±)-α-Terpineol
	II	Unknown
F-PL-D	I	(±)-Citronellal
	II	Unknown^c^
F-PL-E	I	Myrcene, (*E*)-β-ocimene
	II	Unknown^c^
F-PL-F	I	(±)-1-Octen-3-ol
	II	3-Octanone

^a^Only the compounds showing clear differences in the size of the spikes elicited by them are listed here in the ‘active compound’ column, although some other compounds could elicit responses from the corresponding ORNs, as shown in Table 4

^b^The presence of multiple sensory neurons in these types of sensilla was not confirmed in this study

^c^Active compound for this type of sensory neuron was not found in this study although the presence of the neuron was clear by distinct group of spikes

^†^Diketone: 4-Methylheptane-3,5-dione; RR: (4*R*,5*R*)-5-Hydroxy-4-methylheptan-3-one; RS: (4*R*,5*S*)-5-Hydroxy-4-methylheptan-3-one; SR: (4*S*,5*R*)-5-Hydroxy-4-methylheptan-3-one; SS: (4*S*,5*S*)-5-Hydroxy-4-methylheptan-3-one

### ORNs Responsive to Pheromone-Related Compounds

**Fig. 3:**
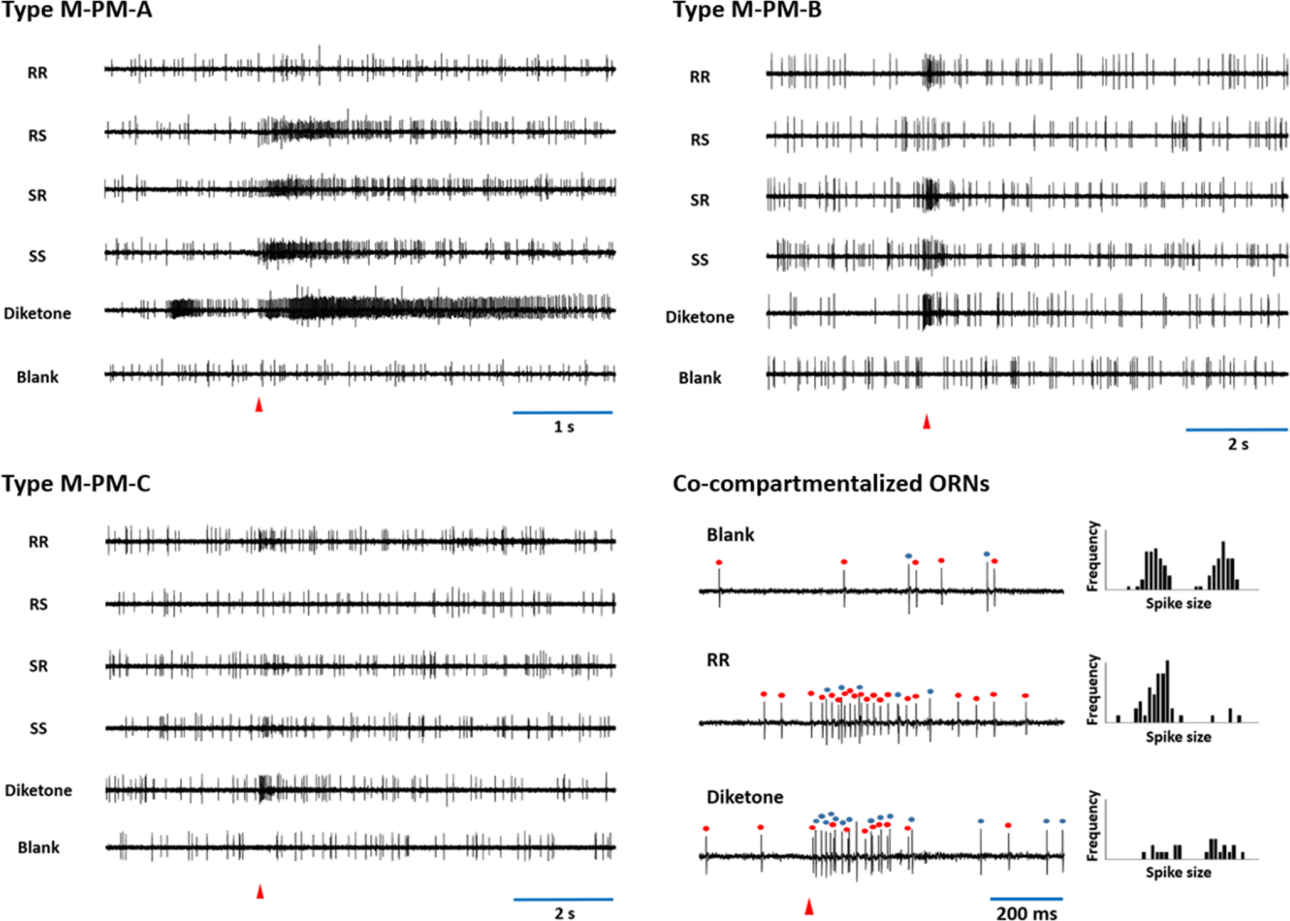
Responses of olfactory receptor neurons (ORNs) housed in three different types of sensilla in male *Sitona discoideus* to the pheromone-related compounds, 4-methylheptane-3,5-dione (diketone), and the four stereoisomers (RR, RS, SR and SS) of 5-hydroxy-4-methylheptan-3-one. Red triangles indicate stimulation for 0.1 s. The presence of two different ORNs co-compartmentalized in type M-PM-C sensilla was verified with the presence of two distinct groups of action potentials with different spike sizes, respectively (bottom right: Histogram distributions display two groups of action potentials, each of which, marked with different colors and responsive to the RR isomer and diketone, respectively, could be clearly distinguished in the spike trains)

The diketone (4-methyl-3,5-heptanedione) and the four stereoisomers (*RR*, *RS*, *SR* and *SS*) of sitophilure, 5-hydroxy-4-methylheptan-3-one (Fig. 1), elicited strong responses in both male and female lucerne weevils. Three different types of sensilla containing ORNs responsive to pheromone-related compounds were identified in the males and females, respectively (Table 3; Fig. 3). Each type of sensillum exhibited a distinct response spectrum to the five pheromone-related compounds tested, in which 4-methyl-3,5-heptanedione elicited the strongest responses and the *RR* isomer of 5-hydroxy-4-methyl-3-heptanone the weakest overall response scores (Table 3). One type of sensillum in males (M-PM-A), the most abundant type of pheromone-related sensilla, and two types of sensilla in females (F-PM-A and F-PM-B) displayed the strongest responses to diketone, indicating that more than 60% of pheromone-related sensilla belonged to these types (Table 3). In contrast, only one type of sensillum in females (F-PM-C) exhibited fair responses to the *RR* isomer of 5-hydroxy-4-methyl-3-heptanone, whereas all other types of pheromone-related sensilla showed only weak or no responses to this compound (Table 3). On the other hand, the three other isomers, *RS*, *SR* and *SS*, of 5-hydroxy-4-methyl-3-heptanone elicited relatively strong responses from M-PM-A and F-PM-A sensilla (Table 3). One type of sensillum in males, M-PM-C, exhibited weak responses only to the *RR*-isomer (Table 3). The response profiles across five pheromone-related compounds were similar between M-PM-A sensilla in males and F-PM-A sensilla in females, whereas the responsiveness of F-PM-B sensilla appeared to be female-specific (Table 3).

### ORNs Responsive to Plant Volatile Compounds

All the ORNs identified in the 143 plant-volatile responsive sensilla exhibited a high degree of specialization in their responsiveness, showing responses only to a narrow range of plant volatile compounds (Table 4). Overall, five different types (M-PL-A ~ E) of sensilla containing ORNs responsive to plant volatile compounds were identified in male *S. descoideus* and six different types (F-PL-A ~ F) of sensilla in the females (Table 4). Among 42 plant volatiles tested, 27 compounds elicited significant responses from the ORNs in these sensilla (Table 4). Some ORNs in four types of sensilla displayed mild responses to hexane solvent control, although none of them showed noticeable responses to hexane used as a test stimulus after it had been dissolved in mineral oil (Table 4). The responses of these plant-volatile responsive ORNs to 15 other compounds were not significantly different from the responses to the control solvent.

Approximately 58% (M-PL-A and M-PL-B) of plant-volatile responsive sensilla in males and 45% (F-PL-A and F-PL-B) in females contained ORNs that were highly sensitive to green leaf volatiles (GLVs), exhibiting strong responses only to some of the nine green leaf volatiles tested (Table 4). These GLV-specific sensilla showed strong responses to green leaf alcohols, and one type in males (M-PL-A) and one type in females (F-PL-A) showed relatively strong responses to an aldehyde (*E*)-2-hexenal (*E*2–6:Ald), whereas two six-carbon acetates (hexyl acetate and (*Z*)-3-hexenyl acetate (*Z*3–6:OAc)) and a seven-carbon ketone (2-heptanone) elicited very weak responses only in some of these sensilla (Table 4). The response profiles of ORNs were similar between two types of GLV-specific sensilla in males and those in females (Table 4). Overall, the GLV-specific sensilla in males exhibited stronger responses to the six-carbon alcohols than those of females, and three unsaturated six-carbon alcohols, (*E*)-2-hexenol (*E*2–6:OH), (*Z*)-2-hexenol (*Z*2–6:OH), and (*Z*)-3-hexenol (*Z*3–6:OH), elicited the strongest responses from the GLV-specific sensilla in the males.

Among the plant-volatile responsive ORNs, approximately 42% of sensilla identified in males (M-PL-C, M-PL-D and M-PL-E) and 55% in females (F-PL-C, F-PL-D, F-PL-E and F-PL-F) contained ORNs responsive to some of 18 plant volatile compounds that are not GLVs, in which each type of sensillum exhibited a distinct response spectrum to a narrow range of volatile compounds (Table 4). Among these seven types of sensilla, two types in males (M-PL-D and M-PL-E) and two types in females (F-PL-E and F-PL-C) responded to similar overlapping active compounds, respectively (Table 4). In contrast, one type in males (M-PL-C) and two types in females (F-PL-D and F-PL-F) exhibited sex-specific response spectra (Table 4). In particular, a type of female-specific sensillum, F-PL-F, showed strong, exclusive responses to 1-octen-3-ol and 3-octanone (Table 4). Among 18 non-GLV olfactory-active compounds, three compounds, myrcene, (*E*)-β-ocimene and 2-phenylethanol, elicited strong responses from the ORNs in two types of male-specific sensilla (M-PL-D and M-PL-E), whereas two compounds, (±)-citronellal and (±)-linalool, elicited strong responses in two types of female-specific sensilla (F-PLC and F-PL-D) (Table 4).

### Co-Compartmentalized ORNs

The number of co-compartmentalized ORNs in the same sensillum and corresponding active compounds for each ORN could be determined in approximately 60% of the sensilla examined in this study by comparing the size of action potentials generated in each sensillum. In the rest of the sensilla examined, however, the number of ORNs in a sensillum could not be determined since the size of action potentials was not clearly distinguishable. Among 17 types of sensilla identified, 14 types of sensilla displayed the presence of two co-compartmentalized ORNs (Table 5). The co-compartmentalized ORNs in the same sensillum exhibited exclusive responses to either pheromone-related compounds or plant volatile compounds, and no sensilla were found to co-compartmentalize both ORNs responsive to pheromone-related compounds and ORNs responsive to plant volatile compounds (Table 5). In pheromone-related compound-responsive sensilla, one type in males (M-PM-A) and one type in females (F-PM-A) co-compartmentalized two ORNs, each of which exhibited strong responses to diketone and the *SR*-isomer, and the *RS*- and *SS*-isomers, respectively (Table 3, Table 5). An ORN showing relatively weak, but specialized, responses to the *RR*-isomer was co-compartmentalized with another ORN displaying weak responses to diketone in male-specific M-PM-C sensilla (Tables 3 and 5; Fig. 3). Similarly, an ORN showing relatively weak, but specialized, responses to the *RS*-isomer was co-compartmentalized with another ORN displaying strong responses to diketone and the *SR*-isomer in female-specific F-PM-B sensilla (Tables 3 and 5).

**Fig. 4:**
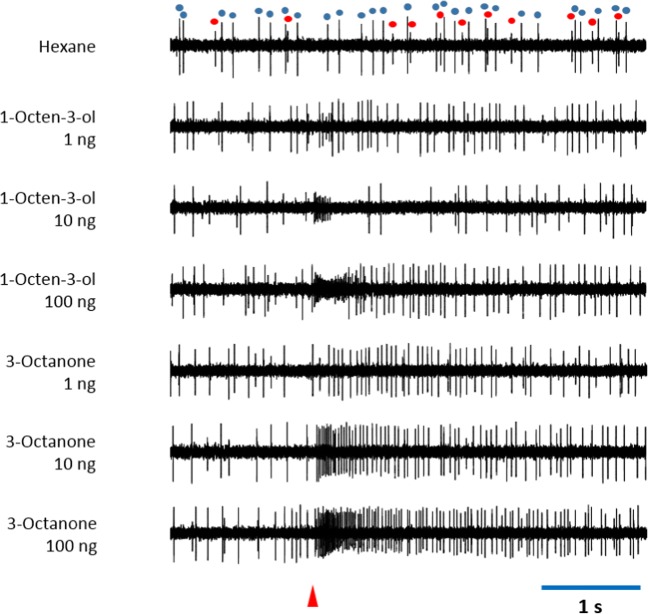
Responses of two olfactory receptor neurons (ORNs) co-compartmentalized in type F-PL-F sensilla in female *Sitona discoideus* to three different doses of 1-octen-3-ol and 3-octanone, demonstrating that these ORNs are highly sensitive to the corresponding stimuli. The ORN generating small spikes (red dots) was exclusively responsive to 1-octen-3-ol, whereas the ORN generating large spikes (blue dots) was exclusively responsive to 3-octanone. Red triangle indicates stimulation for 0.1 s

Ten types of plant-volatile responsive sensilla, five in males and five in females, contained at least two ORNs in the same sensillum (Tables 4 and 5). Among them, two co-compartmentalized ORNs in each of two types of sensilla exhibited specialized responses to a narrow range of compounds. Type M-PL-A sensilla in males and type F-PL-A sensilla in females contained two ORNs, respectively, one specialized for Z2–6:OH, *E*2–6:OH and *E*3–6:OH and the other for *E*2–6:Ald (Table 5). However, only one ORN exhibited distinct responses to a narrow range of compounds tested, whereas the other ORN did not show any responses to the test compounds, in eight other types of sensilla displaying the presence of two co-compartmentalized ORNs (Table 5). Type F-PL-F sensilla in females, exhibiting exclusive responses to 1-octen-3-ol and 3-octanone, appeared to co-compartmentalize two different ORNs, one responsive to 1-octen-3-ol while the other was responsive to 3-octanone (Table 5). These two ORNs displayed very high sensitivity to 1-octen-3-ol and 3-octanone, showing consistent responses to these compounds at the 1-ng level (Fig. 4).

## Discussion

### Pheromone ORNs in *Sitona discoideus*

Our study indicates that *S. discoideus* has a set of antennal ORNs with which diketone and four isomers of monoketone can be detected and distinguished with high sensitivity and selectivity. In particular, the presence of ORNs with high sensitivity to diketone in male and female *S. discoideus* suggests that this compound, a pheromone in *S. lineatus* (Blight et al. 1984, 1991), is likely to play a role as an aggregation pheromone in this species. This is a different response from that with a sibling species, the clover root weevil, *S. obsoletus* in which only males responded to the compound (Park et al. 2013). Interestingly, both sexes of both species responded strongly to all isomers of 5-hydroxy-4-methylheptan-3-one, except the *RR*-isomer which elicited a weak response from one type of sensillum. The discrimination by *S. discoideus* females between stereoisomers of the compound indicates that these isomers have some biological importance for this species. The olfactory role of the *RS* and *SS* isomers appears to be different from those of diketone and two other monoketone isomers, since they are detected by different ORNs co-compartmentalized in the same sensillum. The *RR* isomer may also have a distinct olfactory role in the pheromonal communication in *S. discoideus,* since this compound is detected by a specialized ORN, although the responsiveness of this ORN to the *RR* isomer is relatively weak.

The presence of sex-specific sets of specialized ORNs for different pheromonal compounds implies that male *S. discoideus* can discriminate between diketone and the *SR* isomer, the *RS* and *SS* isomers, and the *RR* isomer and others, and female *S. discoideus* can distinguish between diketone and the *SR* isomer, the *RS* and *SS* isomers, and the *RS* isomer and others. The presence of the *RR*-isomer-sensitive ORN in males and the *RS*-sensitive ORN in females suggests that the *RR* isomer and the *RS* isomer have sex-specific behavioral roles, respectively, in *S. discoideus*. The lack of specialized ORNs for 4-methyl-3,5-heptanedione in *S. obsoletus* females may indicate that this species does not use this compound as an aggregation pheromone (Park et al. 2013). Instead, one or more stereoisomers of 5-hydroxy-4-methyl-3-heptanone may be used as pheromone compounds in *S. obsoletus*, since they can differentiate between the syn- and anti-diastereomers of this compound (Park et al. 2013). The latter appears to be true for male *S. obsoletus*, as the males can also discriminate between the diketone and the monoketone as well as between the anti-enantiomers (*RR* and *SS*).

### Plant Volatile ORNs in *Sitona discoideus*

**Fig. 5:**
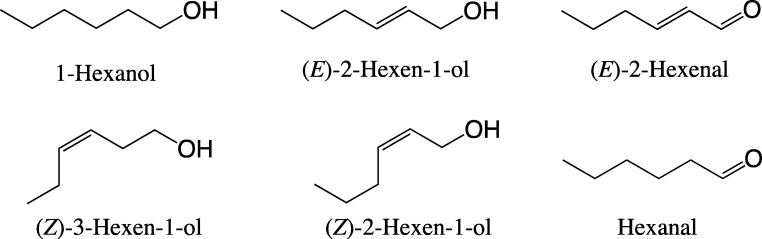
The structures of six green leaf volatiles that elicited strong responses from the antennal olfactory receptor neurons (ORNs) in both sexes of *Sitona discoideus*

Our results indicate that highly sensitive ORNs specialized for detecting plant volatile compounds are present in the antennae of male and female *S. discoideus*. All the ORNs responsive to plant volatile compounds exhibited narrow response spectra, suggesting that these compounds can be discriminated from each other with high precision in *S. discoideus*. A large proportion (58% in males and 45.1% in females) of plant volatile-responsive ORNs in *S. discoideus* are specialized for green leaf volatiles (GLVs) (Fig. 5). All the sensilla housing these GLV-sensitive ORNs exhibited highly sensitive and selective responsive to six-carbon aliphatic alcohols such as *E*2–6:OH, *Z*2–6:OH, and *Z*3–6:OH, and one of two co-compartmentalized ORNs in one type of sensillum in males and females, respectively, elicited highly selective responses to *E*2–6:Ald, suggesting that the alcohols and aldehyde are likely to be key GLV compounds in the host location and recognition in *S. discoideus*. This is supported by earlier reports that *E*2–6:OH, *Z*3–6:OH, and *E*2–6:Ald are major volatile emanations of lucerne (Tava and Pecceti 1997; Landon et al. 1997). However, it is unclear whether these compounds are directly involved with any female-specific behavior of *S. discoideus* such as oviposition, since both male and female *S. discoideus* contain these GLV-specialized ORNs.

The ability to detect GLVs in both sexes of *S. discoideus* and *S. obsoletus* (Park et al. 2013) appears to be natural, as it can be used for host plant detection and also for aggregation around lucerne or clover. *Z*3–6:OH, *Z*2–6:OH and *Z*2–6:Ald have been reported as prominent plant volatiles in both lucerne and red clover. Although these compounds are common green leaf volatiles, the relative composition of different volatiles could form a signature odor profile for these host plants. This hypothesis is supported by the co-compartmentalization of two separate neurons in the same sensillum. The olfactory sensory system for GLVs appears to be similar between two close species, *S. discoideus* and *S. obsoletus*, in the sense that monounsaturated six-carbon GLV alcohols and *E*2–6:Ald are exclusively detected by a set of specialized ORNs (Park et al. 2013). However, there is a difference in the organization of ORNs between two species. Unlike *S. obsoletus,* which shows the presence of sensilla containing two ORNs, one specialized for GLVs and the other specialized for non-GLV plant volatile compounds (Park et al. 2013), *S. discoideus* contain no such olfactory sensilla.

**Fig. 6:**
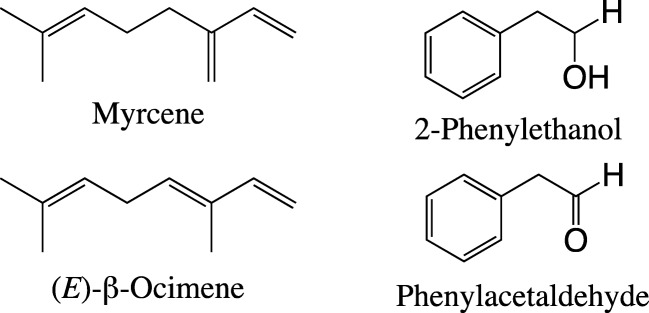
The structures of four plant volatile compounds that elicited stronger responses from the antennal olfactory receptor neurons (ORNs) in male than in female *Sitona discoideus*

**Fig. 7:**
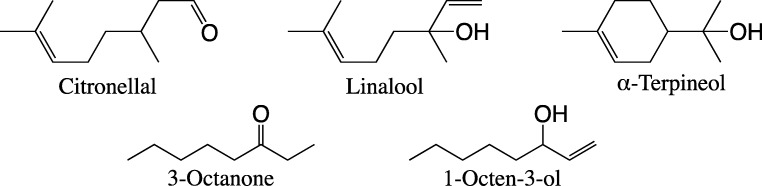
The structures of five plant volatile compounds that elicited stronger responses from the antennal olfactory receptor neurons (ORNs) in female than in male *Sitona discoideus*

Our results also showed that highly sensitive and selective ORNs for non-GLV plant volatile compounds are present in the antennae of *S. discoideus*, and the corresponding olfactory active compounds for these ORNs were clearly different between males and females. For example, male antennae contain specialized ORNs for myrcene, (*E*)-β-ocimene, 2-phenylethanol and phenylacetaldehyde (Fig. 6), whereas female antennae contain specialized ORNs for (±)-citronellal, (±)-linalool, 1-octen-3-ol and 3-octanone (Fig. 7). Studies indicated that four compounds ((*E*)-β-ocimene, 2-phenylethanol, (±)-linalool and 3-octanone) among these seven compounds are produced by lucerne, a main host plant of *S. discoideus* (Landon et al. 1997; Tava and Pecetti 1997). The plant volatiles (±)-linalool, (±)-α-terpineol and (±)-citronellal, eliciting strong responses in the antennal ORNs of female *S. discoideus*, also elicited strong responses from the antennal ORNs in females of a sibling species *S. obsoletus* (Park et al. 2013). The profile of plant volatile ORNs in *S. discoideus* appears to be different from that of another *Sitona* species, *S. humeralis*, since ORNs highly responsive to benzaldehyde, a lucerne flower volatile, are present in this species (Lohonyai et al. 2019), whereas *S. discoideus* did not appear to have such ORNs in our study.

Our study indicated that *S. discoideus* and *S. obsoletus* olfactory sensory systems for non-GLV plant volatile compounds have similarities, but also some species-specific features. Male *S. obsoletus* does not appear to have ORNs specialized for myrcene and (*E*)-β-ocimene, whereas the females of both *S. discoideus* and *S. obsoletus* can detect the oxygenated monoterpenoids linalool, α-terpineol and citronellal (Park et al. 2013). Therefore, these compounds may be used as key compounds for host plant recognition, and possibly serve as oviposition cues, too. An interesting observation is that α-terpineol is a repellent for the maize weevil (*Sitophilus zeamais)* (Ndungu et al. 1995)*.* As it is not reported as a volatile of legumes, this compound could be a repellent also for *Sitona* weevils. The ability of *S. discoideus* to detect myrcene, (*E*)-β-ocimene, 2-phenylethanol and phenylacetaldehyde could be of help when weevils migrate to, or from, their estivation sites. Much stronger responses of males to these compounds could indicate that the males, which migrate first, are directed by these compounds, and that the females are later attracted by pheromonal compounds and/or GLV volatiles released by the first-arriving males. Although Goldson et al. (1990) indicated no difference between the sexes in termination of aestivation, differential movement of males dispersing from diapause sites to pea crops has been reported for *S. lineatus* (Landon et al., 1997). The quantification of 24 compounds emitted from clover showed increased emission rates after herbivory by *Spodoptera littoralis* caterpillars (Kigathi et al. 2009). (*E*)-β-Ocimene was the most abundant compound in the emission, which also contained induced amounts of linalool and 1-octen-3-ol. Typically in Lepidoptera, fatty acid amino acid conjugates from saliva are involved in this response to herbivory by volatile upregulation (Yoshinaga et al. 2010). The selective detection of ORNS for these compounds may suggest that these compounds are key host plant cues, and their upregulation from infestation could be detected by the weevils. Two other chemicals, 1-octen-3-ol and 3-octanone, eliciting strong responses in female-specific, specialized ORNs in the antennae of *S. discoideus*, have been found in the emission from clovers (Kicel et al. 2010, Kigashi et al. 2009), in which 1-octen-3-ol appeared to be a characteristic volatile of legumes. It is yet unclear, however, if *S. obsoletus* also have specialized ORN for these compounds, since these compounds have not been tested in this species. It would be interesting to see if these compounds are involved with any sex-specific behavior in *S. discoideus*.

### Co-Compartmentalization of ORNs

Our study shows that the majority of antennal olfactory sensilla in *S. discoideus* contain more than one ORN in the same sensillum. Each of eight different types of olfactory sensilla among ten different types of olfactory sensilla responsive to plant volatile compounds in *S. discoideus* contained an ORN that did not show any responses to 42 plant volatile compounds tested, suggesting that they are highly specialized for a narrow range of volatile compounds, if they are olfactory. It has been suggested that co-compartmentalization of ORNs facilitates the discrimination of closely moving odor filaments, by providing high temporal and spatial resolution (Baker et al. 1998). Likewise, the co-compartmentalization of two ORNs for two key GLVs - the six-carbon alcohol and its corresponding aldehyde - produced by lucerne, suggests that these two GLV compounds might play a key role in the host location and discrimination of *S. discoideus*. In this context, it would be interesting to investigate why two ORNs for 1-octen-3-ol and 3-octanone, specialized for each compound respectively, are co-compartmentalized in the same sensillum, and to understand if they are involved in any female-specific behavior, as they are present only in female antennae.

## Conclusion

Our study showed that *S. discoideus* possess a characteristic set of highly specialized ORNs for pheromone-related compounds and specific plant volatiles. Field observations suggested that olfactory cues are involved in the aestivation flights of *S. discoideus* in summer towards hedge rows (Goldson et al. 1984), and the post-aestivatory return flights in autumn towards lucerne (Frampton 1987). Some of the highly olfactory-active compounds on antennal ORNs may be involved in these processes, as well as in other olfactory communications, in *S. discoideus*. Therefore, examining behavioral activities of the volatile compounds would be worthwhile in developing semiochemicals for this species. There is obviously a need for behavioural studies to validate the role of antennally active compounds which would link the observed electrophysiological patterns with beetle behaviour, preferably including field trials with synthetic blends and effective trap types.
